# Supplementary figures and images for: Learning dynamic cognitive map with autonomous navigation
Source: Front Comput Neurosci. 2024 Dec 11;18:1498160. doi: 10.3389/fncom.2024.1498160 (PMC11668591; doi:10.3389/fncom.2024.1498160)

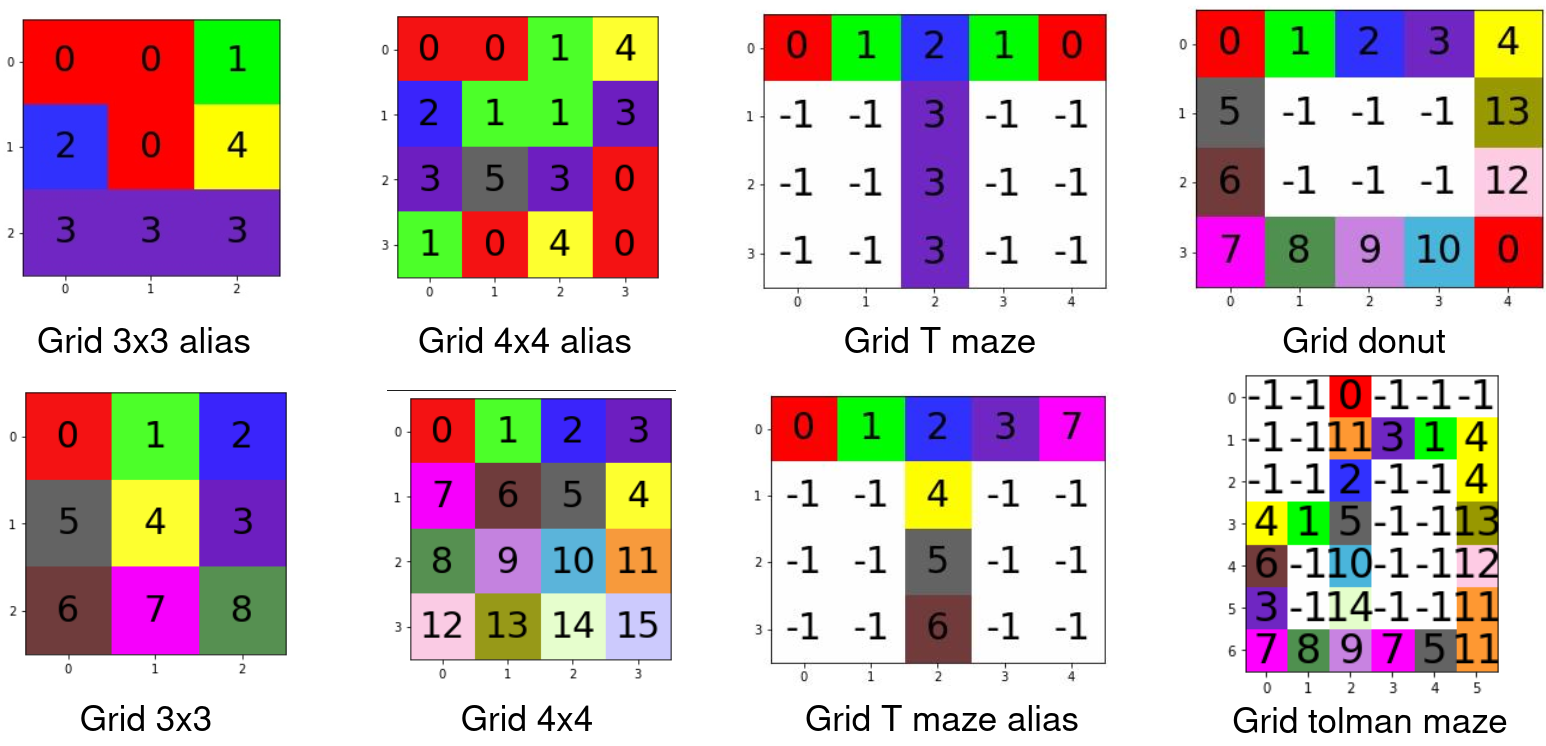

Supplement: Supplementary file 1 [file Presentation_1.zip › envs.png]
